# Short Beam Shear Behavior and Failure Characterization of Hybrid 3D Braided Composites Structure with X-ray Micro-Computed Tomography

**DOI:** 10.3390/polym12091931

**Published:** 2020-08-26

**Authors:** Liwei Wu, Xiaojun Sun, Chunjie Xiang, Wei Wang, Fa Zhang, Qian Jiang, Youhong Tang, Jia-Horng Lin

**Affiliations:** 1 Tianjin and Ministry of Education Key Laboratory for Advanced Textile Composite Materials, Tiangong University, Tianjin 300387, China; wuliwei@tiangong.edu.cn; 2 Innovation Platform of Intelligent and Energy-Saving Textiles, School of Textile Science and Engineering, Tiangong University, Tianjin 300387, China; sunxiaojun36501@163.com (X.S.); ww15620515730@163.com (W.W.); jhlin@fcu.edu.tw (J.-H.L.); 3 3D Composites Division, Nanjing Fiberglass Research & Design Institute Co., Ltd. Jiangsu, Nanjing 210012, China; xiangchunjie688@163.com; 4 Beijing Key Laboratory of Civil Aircraft Structures and Composite Materials, Beijing Aeronautical Science & Technology Research Institute of COMAC, Beijing 102211, China; zhangfa@comac.cc; 5 Institute for NanoScale Science and Technology, College of Science and Engineering, Flinders University, Adelaide 5042, Australia; youhong.tang@flinders.edu.au; 6 Laboratory of Fiber Application and Manufacturing, Department of Fiber and Composite Materials, Feng Chia University, Taichung 40724, Taiwan

**Keywords:** hybrid, braided composites, short beam shear, micro-CT

## Abstract

Three-dimensional braided composite has a unique spatial network structure that exhibits the characteristics of high delamination resistance, damage tolerance, and shear strength. Considering the characteristics of braided structures, two types of high-performance materials, namely, aramid and carbon fibers, were used as reinforcements to prepare braided composites with different hybrid structures. In this study, the longitudinal and transverse shear properties of 3D braided hybrid composites were tested to investigate the influences of hybrid and structural effects. The damage characteristics of 3D braided hybrid composites under short beam shear loading underwent comprehensive morphological analysis via optical microscopy, water-logging ultrasonic scanning, and X-ray micro-computed tomography methods. It is shown that the shear toughness of hybrid braided composite has been improved at certain degrees compared with the pure carbon fiber composite under both transverse and longitudinal directions. The hybrid braided composites with aramid fiber as axial yarn and carbon fiber as braiding yarn exhibited the best shear toughness under transverse shear loading. Meanwhile, the composites with carbon fiber as axial yarn and aramid fiber as braiding yarn demonstrated the best shear toughness in the longitudinal direction. Due to the different distribution of axial and braiding yarns, the transverse shear property of hybrid braided structure excels over the longitudinal shear property. The failure modes of the hybrid braided composite under the two loading directions are considerably different. Under transverse loading, the primary failure mode of the composites is yarn fracture. Under longitudinal loading, the primary failure modes are resin fracture and fiber slip. The extensive interfacial effects and the good deformation capability of the hybrid braided composites can effectively prevent the longitudinal development of internal cracks in the pattern, improving the shear properties of braided composites.

## 1. Introduction

The yarns of 3D braided composites intertwined with one another in space to form a highly integrated 3D mesh structure can fundamentally overcome the problems of poor adhesion between layers and easy delamination, and thus, improve the structural integrity and mechanical properties of composites along the thickness direction [1,2]; such composites have been widely used in the aerospace, military, marine, automobile, and other fields [3,4,5]. With the continuous expansion of the understanding and application of braided composites, the demand for high-performance 3D braided composites is also increasing. One of the key problems of 3D braided composites is their high degree of anisotropy, and these composites frequently bear multidirectional loads in actual application processes [6]. Carbon fiber is widely used in the field of composite materials because of its high tensile strength. However, there are also some shortcomings in carbon fiber reinforced composites, such as poor impact resistance, shear resistance and energy absorption [7]. Therefore, improving the comprehensive mechanical properties of materials by designing in accordance with the structural characteristics of 3D braided composites is highly significant.

At present, 3D braided composites are mostly made of carbon fiber and other fibers with a single component as reinforcement. Although their performance is stable, their function is singular; moreover, performance, cost, brittleness, toughness, damage, lifetime, and other factors cannot be considered simultaneously. As a solution, many researches have focused on hybrid fibrous composites, with ductile fibers such as basalt, aramid, and glass fibers been added to carbon fiber reinforced composites to enhance their damage tolerance. Hybrid braiding is one of the most effective methods for modifying the properties of 3D braided composites [8]. Hybrid braided composites typically refer to materials with two or more fibers added to the resin [9]. The advantage of hybrid braided composites is that they improve the properties of composites by compensating for the disadvantages of one fiber through fiber hybridization. Zheng et al. used digital image correlation technology to study the influence of braided and hybrid structures on the tensile properties of carbon/Kevlar hybrid 3D braided composites; they found that the 3D five-directional (3D5d) braided composites with carbon fiber as axial yarn exhibited higher tensile strength and modulus [10]. Wu et al. studied the influence of braided and mixed braided structures on the low-speed response of carbon/aramid hybrid braided 3D5d composite via micro and ultrasonic nondestructive testing; they determined that the composite with aramid fiber as axial yarn and carbon fiber as braided yarn achieved the best impact resistance [11]. Zhang et al. investigated the progressive bending damage of 3D5d braided composite by using acoustic emission technology and X-ray micro-computed tomography; the results showed that the progressive failure mechanisms of longitudinal and transverse specimens are significantly different [12]. Therefore, introducing a high-performance fiber into 3D5d composite can enable this type of material to achieve balance among various properties, increasing its designability and application range.

Scholars have recently conducted numerous studies on the damage and failure behavior of 3D braided composites under axial tension, compression, bending, fatigue, and impact; meanwhile, the effect of shear progressive damage on 3D braided composites is not yet fully understood at present [13,14,15]. Aswani et al. studied the plane shear properties of Kevlar/basalt fiber-reinforced thermoplastic composites; they determined that the shear modulus, shear strength, and shear failure strain of hybrid braided composites were 6.5–14.9%, 4.3–19.7%, and 3.2–46.7% higher than those of homogeneous composites, respectively [16]. He et al. investigated the properties of the composites at different temperatures by mechanical experiments and finite element method; the results showed that with the increase of temperature, the properties of 3D braided composites decreased, and the failure mode changed from fiber fracture to matrix plastic deformation [17]. Liu et al. proposed an improved double-notch specimen to investigate the shear strength of carbon/carbon (C/C) composites at ultrahigh temperatures [18]. By comparing different types of C/C composites at varying high temperatures, these authors concluded that the shear strength of C/C composites depends on the microstructure and fiber at room temperature for a volume fraction of shear load. Shear is one of the basic forms of deformation in composites. It refers to the relative dislocation of the cross section of members along the direction of an external force. In engineering, the deformation of components under a load mostly occurs as a combination of several basic deformations (i.e., tensile, compression, shear, torsion, and bending). The short beam shear test is suitable for measuring the shear strength of high-modulus fiber-reinforced polymer composites. Shear load is the primary load form, and different failure types may occur. Scholars have conducted extensive researches on the damage evolution and failure characteristics of textile composites [19]. The damage mechanism of fiber-reinforced composites can be explored from the aspects of transverse microcracks, crack growth, fiber fracture, micro-delamination, and debonding of fibers [20,21,22]. Cui et al. systematically studied the mechanical properties, progressive failure rule, and fracture mechanism of 3D5d braided composites at different braiding angles by using a split Hopkinson pressure bar [23]. On the basis of Murakami–Ohno damage theory, Wang et al. examined the progressive damage process of 3D four-directional braided composites under uniaxial tension by using the yarn deformation element model of entanglement [24]. Zhou et al. used X-ray computed tomography (XCT) to obtain a 3D image of a 3D braided composite tube after an impact test; they studied the effects of impact speed, braiding angle, and number of braiding layers on the damage mechanism [25,26].

The low damage tolerance is one of the major technical barriers that limit the development of composites. In order to overcome the barrier, in consideration of the characteristics of a braided structure, choosing hybrid fibers as reinforcement can maintain its structural advantages and enrich its mechanical properties, such as improving toughness and avoiding brittle fracture. However, hybrid fiber structures will also increase the complexity of failure mechanism analysis. Due to fiber orientation within the braided structure, the longitudinal and transverse properties of 3D braided composites are diverse. In the current work, considering the characteristics of braided structures, two types of high-performance materials, namely, aramid and carbon fibers, were used as reinforcements to prepare braided composites with different hybrid structures. The effects of braided architecture and co-braided hybrid structure on the short beam shear response of the carbon–aramid hybrid braided composites were experimentally investigated under two loading modes. Immersion focused ultrasound imaging, microscopy, and micro-computed tomography (µCT) were used to examine the hybrid effect and damage evolution of the composites for the design and application of the carbon–aramid hybrid 3D braided composite structure.

## 2. Materials and Methods 

### 2.1. Materials and Sample Preparation

Carbon fiber tows (fiber tows, T300-3K, Toray^®^, Tokyo, Japan), tow size: 200 × 2 tex stranding, aramid fiber (aramid fiber, Kevlar49, DuPont^®^, Wilmington, NC, USA), tow size: 158 × 2 tex stranding, and epoxy resin (epoxy resin, TDE-86, Jingdong^®^, Tianjin, China) were used to braid the hybrid braided composites with 3D5d features [27]. Table 1 lists the specific properties of the raw materials.

**Figure 1:**
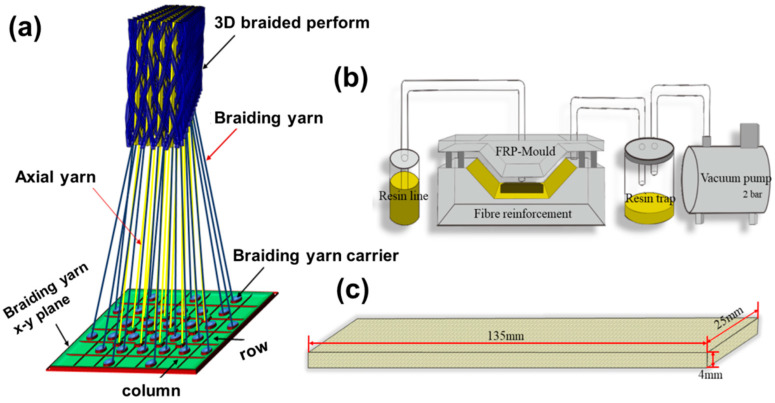
Sample preparation diagrams, (**a**) braiding process; (**b**) RTM process; (**c**) composite dimension.

A four-step 1 × 1 braiding technique was used to braid the 3D5d structure with a rectangular cross section. In contrast with the traditional 3D4d structure, 3D5d has axial yarns aligned along the longitudinal direction of the braided preform. The sample specifications are provided in Table 2. The composites were prepared via resin transfer molding [28]. Under an injection pressure of 0.2–0.3 MPa and a temperature of 130 °C, resin was injected into the sealing mold with prefabricated parts. After resin was completely impregnated into the preforms, the preforms were cured in an oven. The preparation process and the sample dimensions are presented in Figure 1. The specifications of the three types of composites are listed in Table 2, where “C” represents carbon fiber, “K” represents aramid fiber, “a” represents axial yarn, and “b” represents braiding yarn.

### 2.2. Tests

**Figure 2:**
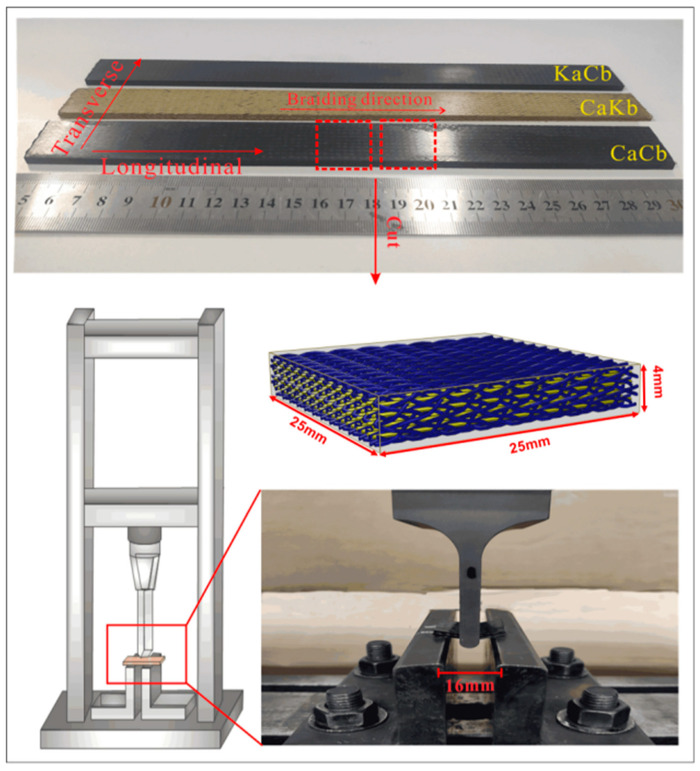
Short beam shear test specimen and testing machine.

For the short beam shear tests, the standard test method (ASTM D2344) was adopted. To study the overall mechanical properties of the 3D braided composite material, the geometric size of a test sample was set as 25 mm × 25 mm × 4 mm, and all the tests were conducted on a universal testing machine (Shimadzu AG-10KNE, Kyoto, Japan) at room temperature (Figure 2). The direction in which the axial yarn is perpendicular to the upper roller is defined as transverse, and the direction in which the axial yarn is parallel to the upper roller is defined as longitudinal. The specimens were tested transversely and longitudinally, and the head pressure velocity was 1 mm/min. Each test result was derived from the average value of three repeated experiments, and the load–displacement curve and the maximum load can be obtained. Furthermore, shear strength (S) can be calculated using

(1) $$S=\frac{3P_{\max}}{4bt}$$

where *b* (mm) and *t* (mm) are the width and thickness of the specimens, respectively. *P*_max_ is the measured maximum load [29].

### 2.3. Water-Logging Ultrasonic Scanning Testing

The ultrasonic testing method based on the principle of sound transmission is a common analytical tool for measuring the internal damage of composite materials. When sound waves hit damaged areas in a composite medium, scanned images with different colors can be formed and damage types can be recorded intuitively. In this study, an immersion ultrasonic C-scan detector (UPK-T18, Physical Acoustics Corporation, Princeton Junction, NJ, USA) was adopted to obtain the ultrasonic C-scan image of the 3D braided composite material after shear test. An ultrasonic ACIS-03 system was used for date acquisition and U-View software was used for analysis and imaging. To reduce the influence of the shape of the final failed specimen on data measurement, the damaged specimen was first restored to the plane state, placed in a water tank, and a focused ultrasonic probe was used. The central ultrasonic frequency was 5 MHz, wafer diameter was 12.7 mm, and focal length was 50.8 mm. When different material interfaces were encountered, the probe simultaneously functioned as ultrasound transmitter and receiver. Ultrasonic energy was reflected, and the computer outputted the C-scan results, which showed the internal damage distribution.

### 2.4. Micro-CT Image Acquisition

With the improvement in spatial resolution, the decrease in acquisition time, and the increase in the availability of computed tomography (CT) systems, µCT imaging has become one of the most commonly used nondestructive detection methods. This technique cannot only determine the internal structure and damage characteristics of materials without damaging the integrity of materials, but can also provide highly accurate 3D fiber structure, manufacturing defects, and damage accumulation [30]. In the current study, µCT analysis of the internal damage of the samples was performed on high resolution 3-D X-ray microscope (Zeiss Xradia 510 Versa, Carl Zeiss AG, Oberkochen, Germany). A sample was placed on a rotating fixture, and the spatial resolution was set as 20.668 μm. Then, a current of 132 μm and a voltage of 50 kV were applied. During 3D X-ray image acquisition, the sample was rotated 360° along the *y*-axis to scan it layer by layer. The total time of each scan was approximately 1.2 h. Dragonfly software was used to slice and reconstruct the 2D image. The structural characteristics and material properties of the reconstruction model are identical to those of the 3D braided composite. The 3D reconstruction model of the sample was generated using the same software (i.e., Dragonfly).

## 3. Results and Discussions

### 3.1. Mechanical Behavior

**Figure 3:**
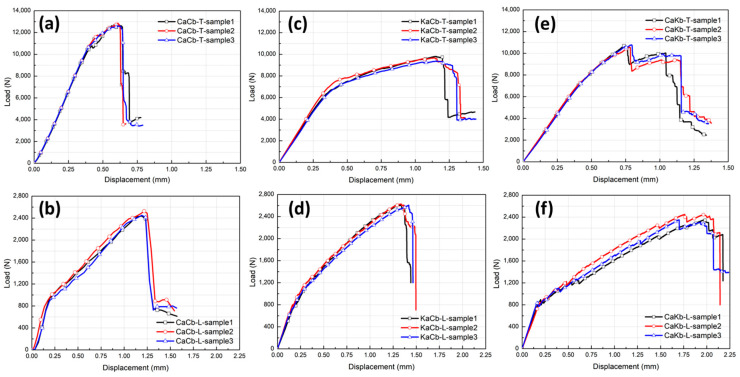
Shear load–displacement curves of samples under transverse loading (**a**) CaCb-T, (**c**) KaCb-T, (**e**) CaKb-T and longitudinal loading (**b**) CaCb-L, (**d**) KaCb-L and (**f**) CaKb-L.

Figure 3 shows the load–displacement curves of three types of 3D5d braided composite specimens under transverse and longitudinal loadings. Here, each curve can provide a comprehensive depiction of damage initiation and growth, along with changes in specimen stiffness. In the beginning, all the curves exhibit a linear increase. However, as shear loading continues, the curve trends of the six types of samples evidently differ. This result indicates that the shear properties of the 3D braided composites with different hybrid structures significantly vary, and the internal structure and yarn properties of the composites exert a significant effect on the failure mechanism.

The curve of the CaCb specimens exhibits good linearity under transverse loading. As displacement reaches 0.37 mm, the slope of the curve decreases gradually and a slight jitter is observed, indicating that damage has occurred in the interior or the surface of the specimens due to matrix cracking and fiber fracture. Thereafter, the load drops sharply, indicating a large number of yarn breaks at this time and exhibiting brittle failure characteristics (Figure 3a). Compared with that of the CaCb samples, the curves of the KaCb and CaKb samples present similar elastic-plastic nonlinear responses, obtaining larger bending deflections and lower maximum loads, and demonstrating a ductile failure feature (Figure 3c,e). For the KaCb samples, modulus is higher in the linear phase of the curve. When displacement reaches 0.33 mm, modulus decreases, signifying initial damage in the material [31]. As plastic deformations and internal damages accumulate, the specimens fail completely once the load reaches the maximum value. From the entire curve, KaCb has an extremely long plateau, indicating that the shear fracture toughness of KaCb is considerably improved compared with that of the pure carbon composite (CaCb). For the CaKb samples, the curve exhibits good linearity at the initial loading, but the slope of the curve decreases slowly after the displacement reaches 0.38 mm. Sudden load drops are observed after the first peak. This condition can be attributed to the occurrence of critical structure damages caused by yarn fracture. Thereafter, load redistribution is observed in the remaining braided composite until the final catastrophic failure occurs [32]. A conclusion can be drawn that incorporating aramid fiber can effectively improve the ductility of 3D braided composites reinforced by pure carbon fiber. Moreover, the hybrid braided composites with aramid fiber as axial yarn and carbon fiber as braiding yarn demonstrate the best shear toughness under transverse shear loading. In the 3D5d braided structure, the braided and axial yarns may exert different influence mechanisms on the mechanical properties of the composite due to the parallel and horizontal arrangement of the axial yarn in the braided structure and the oblique and staggered distribution of the braided yarn [33]. The axial yarn is relatively extended and perpendicular to the loading head; thus, it is the major load-bearing part under transverse shear loading.

Under longitudinal loading, the shear load–displacement curves of the three specimens demonstrate similar nonlinear elastic-plastic responses (Figure 3b,d,f). Compared with that of transverse loading, the maximum load of longitudinal loading on the specimen decreases and failure displacement increases. These results may be attributed to the orientation of the axial and braided yarns in the sample being in the same loading direction, resulting in the insufficient utilization of the shear coordination mechanism of the braided structure. The matrix is the major bearing part during this time. A conclusion can be drawn that the shear properties of the braided composites significantly differ in the longitudinal and transverse directions and that the loading direction and orientation of the yarns inside the material significantly affect the failure mechanism. For the CaCb samples, the linear phase of the curve is shorter. When displacement reaches 0.13 mm, the slope of the curve decreases and jitter occurs. Then, internal damage accumulates until the maximum load is reached and the samples suddenly fail. The trends of the KaCb and CaCb sample curves are consistent, indicating that they have the same damage form. Compared with those of the KaCb and CaCb samples, the curve of the CaKb sample presents a larger shear displacement. This finding showed that the composite material achieves the best shear toughness when carbon fiber is used as axial yarn and aramid fiber is used as braiding yarn.

**Figure 4:**
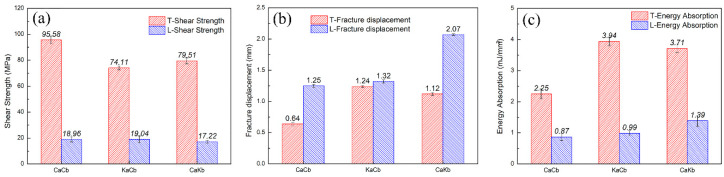
(**a**) Shear strength, (**b**) Fracture displacement and (**c**) Energy absorption of three types of composites under transverse and longitudinal directions.

Figure 4 shows the shear strength, failure displacement, and energy absorption per unit volume under transverse and longitudinal loadings of the three composites. The energy absorbed per unit volume (unit: MJ/mm^3^) is calculated using the area under the load–displacement curve. Each result is the average value of the three samples with an error range of less than 5%. Under transverse loading, the shear strengths of the CaCb, KaCb, and CaKb samples are 95.58, 74.11, and 79.51 MPa; the failure displacements are 0.64, 1.24, and 1.12 mm; and the energy absorption capacities are 2.25, 3.49, and 3.71 MJ/mm^3^, respectively. Compared with the CaCb samples, the KaCb and CaKb samples have 22% and 17% lower transverse shear strength, 94% and 75% higher failure displacement, and 75% and 64% higher energy absorption capacity. A conclusion can be drawn that the shear strength of the hybrid braided sample decreases under transverse loading. Meanwhile, failure displacement and energy absorption capacity are considerably improved. The absorption capacity of a sample is positively correlated with its shear displacement. In addition, the shear strength of KaCb is 7% lower than that of CaKb, but its failure displacement and energy absorption capacity are 10% and 6% higher, respectively. Therefore, the hybrid braided sample KaCb exhibits better shear toughness under transverse loading. The reason why hybrid braided structure of aramid fiber as axial yarn and carbon fiber as braiding yarn exhibited the best shear toughness under transverse loading is that the axial yarn is relatively stretched in the braided structure and perpendicular to the loading direction, which plays the main supporting role under the transverse loading. The aramid fiber, when acted as the axial yarn, exhibits better shear resistance than carbon fiber because its ductility effectively prevents the propagation of internal cracks, which makes the KaCb sample have a longer progressive damage evolution process.

Under longitudinal loading, the shear strengths of the CaCb, KaCb and CaKb samples are 18.96, 19.04, and 17.22 MPa; the failure displacements are 1.25, 1.32, and 2.07 mm; and the energy absorption capacities are 0.87, 0.99, and 1.39 MJ/mm^3^, respectively. The shear strength, failure displacement, and energy absorption of the KaCb sample are extremely close to that of the CaCb sample. The shear strength of the CaKb sample decreases slightly compared with that of the CaCb sample, but its failure displacement increases by 65% and its energy absorption capacity increases by 60%. Thus, the shear toughness of the CaKb sample is better under transverse loading. In addition, the mechanical properties of the transversely and longitudinally loaded samples are significantly different. The shear strength, failure displacement, and energy absorption capacity of the transversely loaded samples are all higher than those of the longitudinally loaded samples.

### 3.2. Damage Morphology Analyses

**Figure 5:**
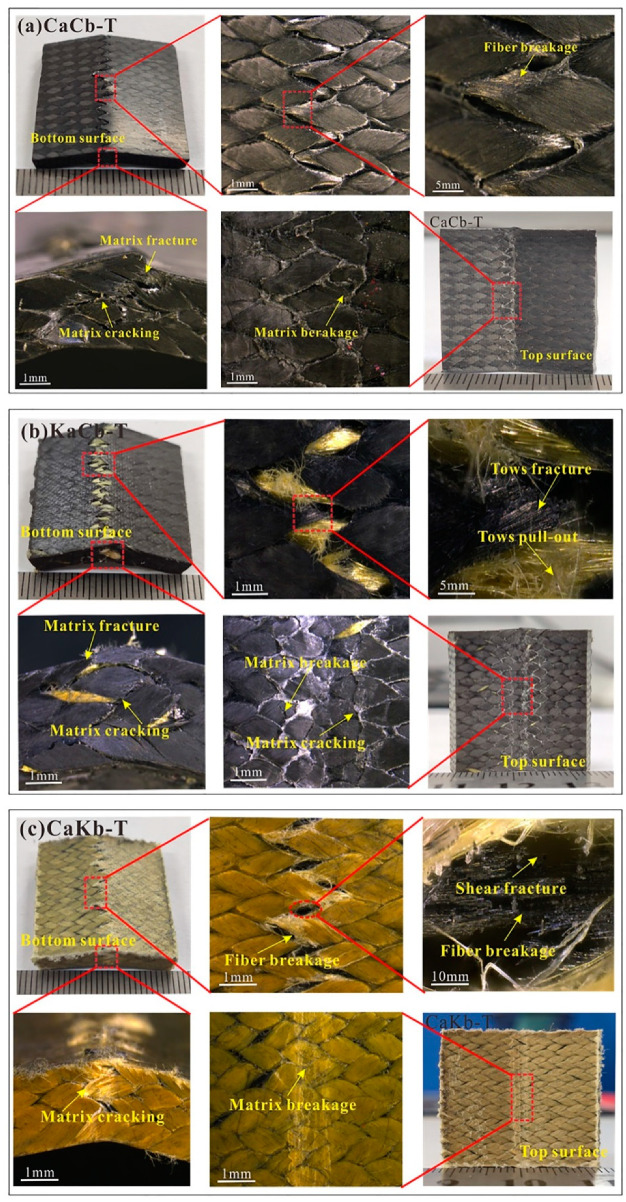
Macro damage morphology of (**a**) CaCb, (**b**) KaCb and (**c**) CaKb under transverse shear loading.

Figure 5 shows the failure damage morphology of CaCb, KaCb, and CaKb under transverse shear loading. The specimens are subject to shear and bending-induced deformations, and macroscopic damage is mostly concentrated in the loading area. The failure modes are primarily matrix cracking, matrix fracture, debonding and delamination of the fiber–matrix interface, fiber breakage, and tow debonding cracks. The CaCb sample is fractured as a whole with a small degree of deformation. The top surface has a kink band formed by the extrusion of the matrix, the sides exhibit matrix cracking and debonding and delamination of the fiber–matrix interface, and the bottom has a matrix fracture that forms a neat fracture. On the basis of the analysis of the load–displacement curve, it can be seen that the brittle failure characteristics of the CaCb specimen are caused by the brittle fracture of the yarn and the resin. The deformation degree of the hybrid braided samples is larger after loading failure. The KaCb specimen (Figure 5b) exhibits severe matrix kink band on the loading surface, matrix cracking on the side, debonding and delamination of the fiber–matrix interface, crack propagation, and tooth fracture formed by the fracture of the braided yarn and resin at the bottom. In addition, the aramid fiber of the KaCb sample is pulled out and debonded but not completely broken. The results show that the aramid fiber in the axial yarn of the KaCb sample demonstrates local gradual fracture failure rather than whole fracture similar to that of the carbon fiber. This finding further proves that aramid fiber plays an important role in transverse shear fracture toughness. For the CaKb sample (Figure 5c), the top surface is extruded by the matrix to form a kink band, and the side surface is cracked by the matrix, resulting in the debonding of the fiber–matrix interface. At the bottom surface, the braided aramid fiber bundle is cracked and pulled out on the loading underside, and brittle fracture of the axial carbon fiber bundle is observed. From the load–displacement curve, the brittle fracture of the carbon fiber in axial yarn may be the cause of the precipice drop of the CaKb specimen under transverse loading.

**Figure 6:**
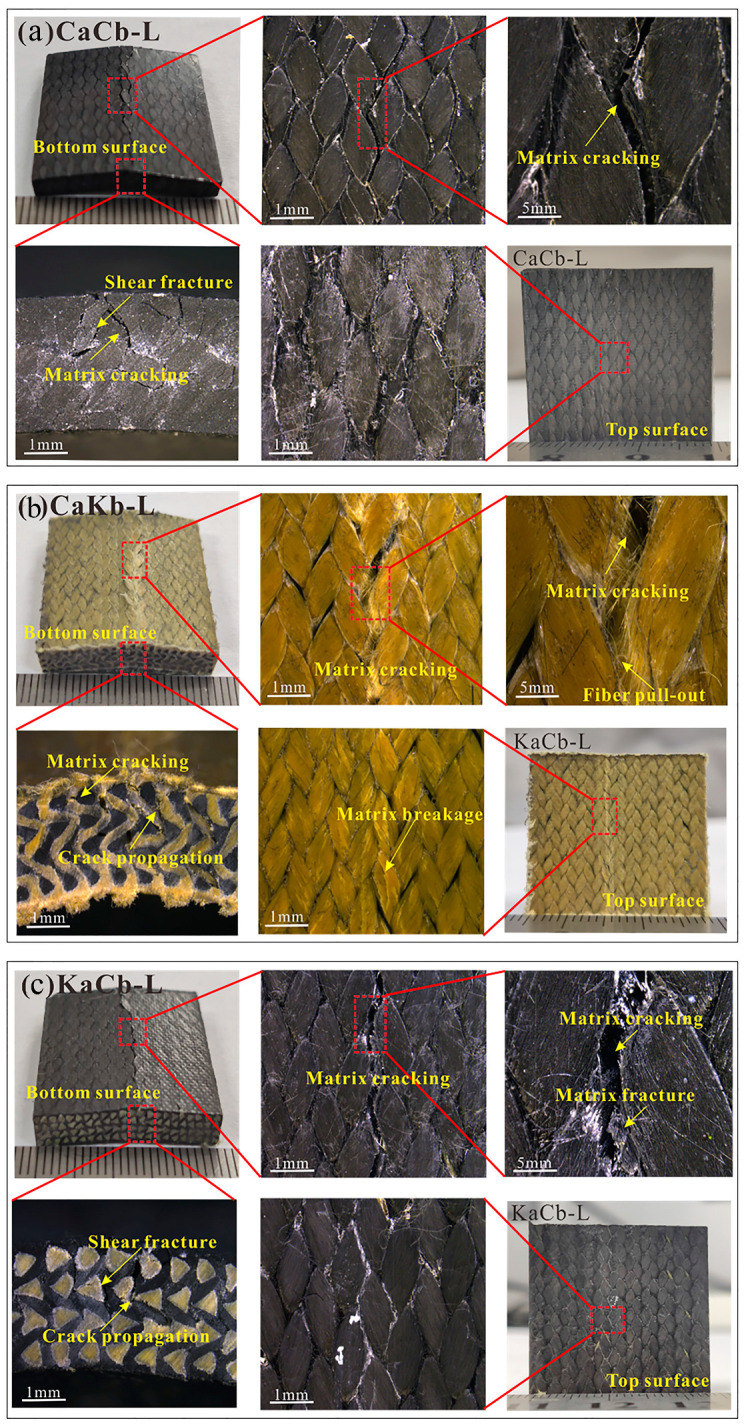
Macro damage morphology of (**a**) CaCb, (**b**) KaCb and (**c**) CaKb under longitudinal shear loading.

Figure 6 shows the damage morphology of CaCb, KaCb, and CaKb under longitudinal shear loading. In contrast with transverse loading, the macro failure of the longitudinal loading is completely dominated by the shear fracture. The damage modes are matrix fracture, matrix cracking, fiber slip, and interface debonding. For the CaCb sample (Figure 6a), the matrix fracture is serious. A neat fracture is formed by resin cracking on the loading bottom, matrix cracking on the side, and the crack extends upward along the loading bottom. The failure of the CaCb specimen is caused by the brittle fracture of the resin. The damage characteristics of the KaCb sample (shown in Figure 6b) are similar to those of the CaCb sample. The cracks formed by the matrix fracture and the matrix crack become larger. The deformation of the CaKb sample is serious after loading, and a slight matrix extrusion occurs on the loading surface, seen in Figure 6c. Cracks are formed on the bottom surface due to the yield of the aramid yarn and the debonding of the matrix. Hence, the aramid fiber of the braided yarn is the reason why the longitudinal shear property of CaKb is higher than those of CaCb and KaCb.

### 3.3. Water-Logging Ultrasonic Imaging Analyses

**Figure 7:**
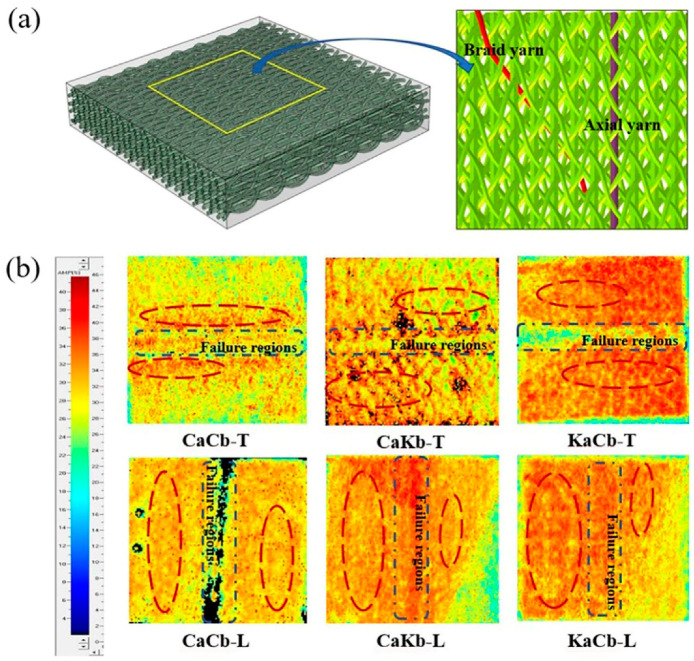
(**a**) 3D spatial interweaving structure in the braided composite; (**b**) ultrasound scanning images of CaCb, KaCb, and CaKb under transverse and longitudinal shear loading.

An optical microscope can only observe the macro damage area of braided composites. For areas without evident damage, ultrasonic nondestructive testing is an effective tool for the qualitative analysis of internal damage difference. In the ultrasonic testing of fiber-reinforced composite materials, the sound beam propagates along the axis of the fiber in one direction; this process cannot only provide the information of substrate interface and defects, but can also detect the location, size, and direction of defects [34]. To investigate intuitively the damage behavior of the specimens, an ultrasonic water-logging system is used to capture the images of whole specimens. Notably, the image designation strips indicate the intensity of the reflected wave. That is, if the color changes from blue to red, then the reflected wave is enhanced, suggesting different damage modes. Figure 7a shows the 3D spatial interweaving structure in the braided composite, which presents the shape distribution of axial and braided yarns in the internal structure. Figure 7b shows the C-scan images of three specimens after failure under transverse and longitudinal loadings. When the red areas in the images are deeper, the degree of internal failure is higher.

The internal braided structures of the three sample materials remain the same. The difference in the C-scan images can identify various damage shapes at the interface between fiber orientation layers [35], reflecting the interface effect caused by different types of fiber components. Under transverse loading, the damage zone of a sample is distributed along the axial and braided yarn paths, indicating the existence of damage in the yarn–resin interface inside the sample. The difference among the C-scan images of the three samples shows that the internal damage band of the samples is closely related to fiber composition and internal arrangement. The damage area of the CaCb sample is concentrated on both sides of the failure area. Meanwhile, the damage area of the hybrid braided specimen extends to the edge. Under transverse loading, the axial yarn carries more load than the braided yarn. When carbon fibers are used as axial yarns, the interfaces among yarns and matrices will be damaged. Meanwhile, the use of aramid fibers as axial yarns will lead to matrix failure, demonstrating that aramid fibers have better interfacial compatibility. Under longitudinal loading, the damage zone is uniform across the matrix. The orientation of the axial and braided yarns in the braided structure is consistent with the loading direction, and the braided yarn and the matrix are the major load-bearing regions. These results show that the damage of the specimen under longitudinal loading is primarily matrix damage. However, when 30% of the received echo amplitude is used as the threshold, the calculated damage area proportions of these types of samples [36], i.e., CaCb, KaCb, and CaKb, under transverse and longitudinal loadings, are 27.0%, 40.2%, and 33% and 21%, 27%, and 34%, respectively. Therefore, the internal damage of the hybrid braided specimen is more than that of the pure carbon braided specimen under the two loading modes. This finding can also explain why the hybrid braided specimen exhibits better shear resistance and energy absorption capacity.

### 3.4. Micro-CT Tomography Analyses

**Figure 8:**
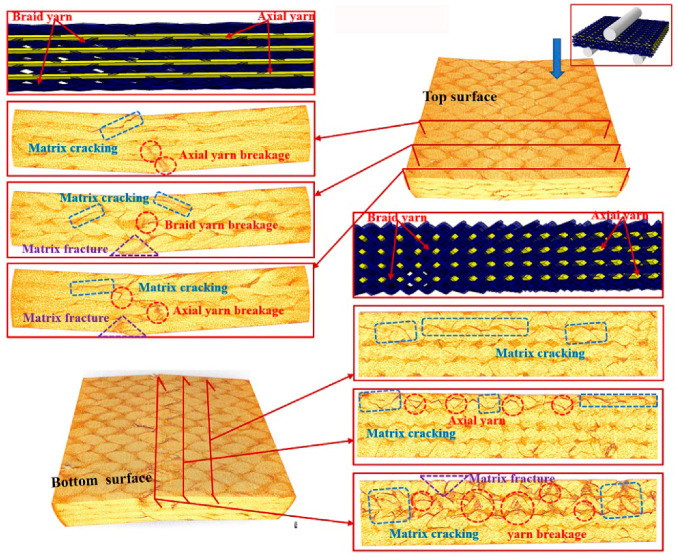
Micro damage morphology of CaCb sample under transverse shear loading.

**Figure 9:**
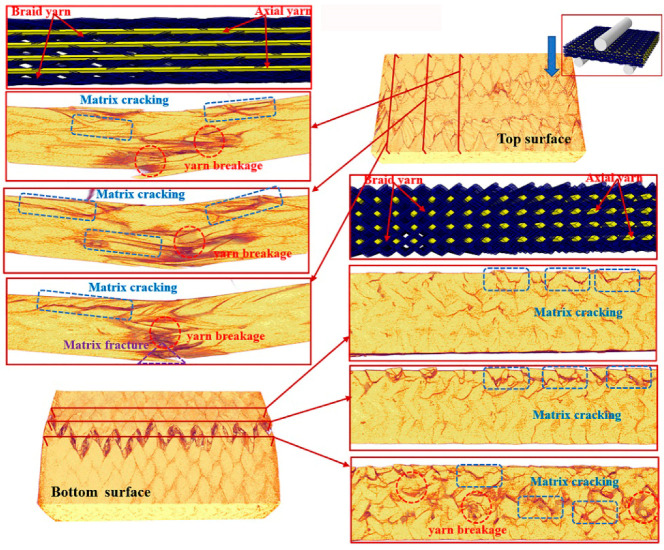
Micro damage morphology of KaCb sample under transverse shear loading.

**Figure 10:**
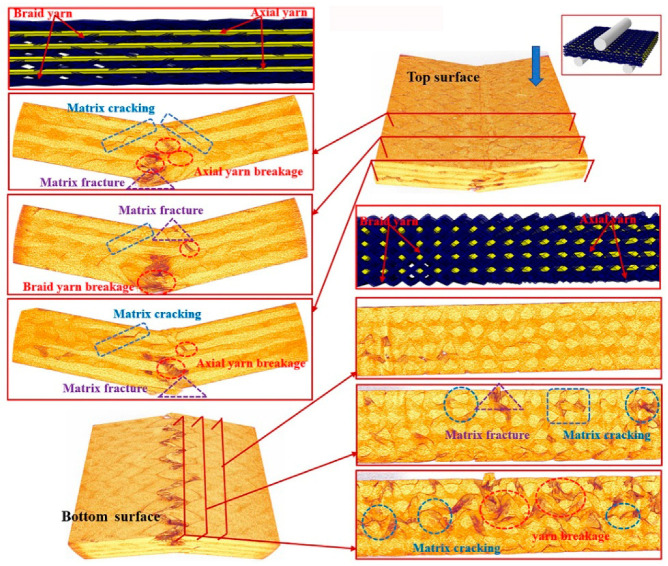
Damage morphology of CaKb sample under transverse shear loading.

**Figure 11:**
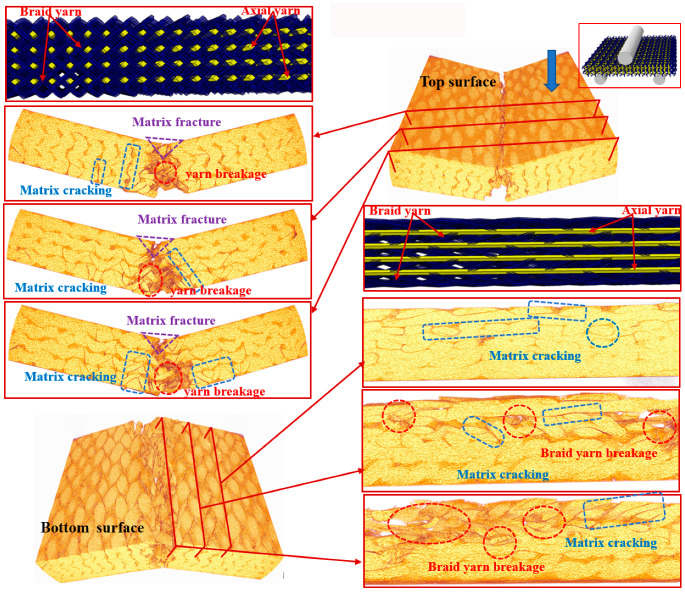
Micro damage morphology of CaCb sample under longitudinal shear loading.

**Figure 12:**
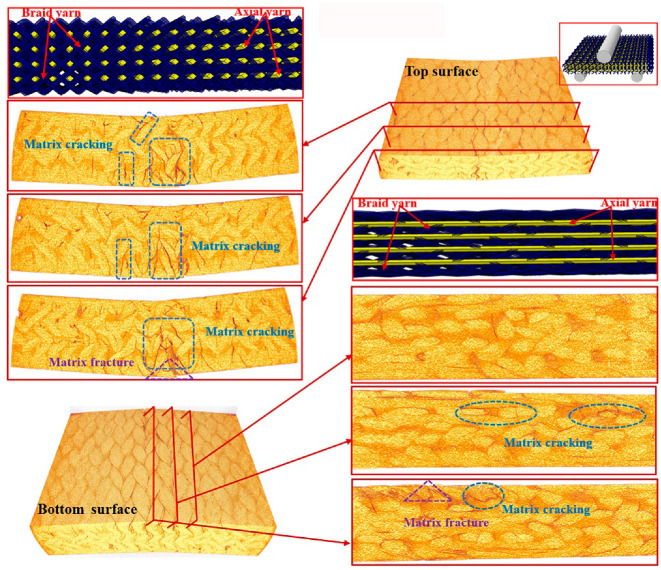
Micro damage morphology of KaCb sample under longitudinal shear loading.

**Figure 13:**
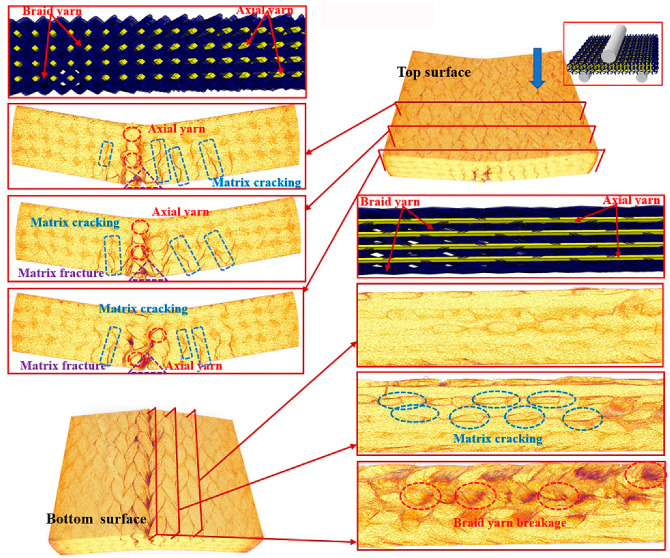
Micro damage morphology of CaKb sample under longitudinal shear loading.

To further understand the underlying damage mechanism of the 3D braided composites at the microstructure level, the reconstructed 3D images and the 2D cross-sectional slices of the ultimate fracture of the three types of samples, which were obtained using µCT, are presented in Figure 8, Figure 9, Figure 10, Figure 11, Figure 12 and Figure 13. The dark red areas in the images correspond to cracks and damages, and the light yellow areas represent higher-density materials, i.e., fiber tows [37]. In the µCT scan of the samples, the complex failure modes of the braided composite, which is concentrated in the loading region, are clearly shown. The major damage modes are matrix cracking, matrix fracture, fiber–matrix interface debonding and delamination, and fiber bundle fracture and cracking. In addition to yarn fracture, the interface crack between the fiber and the matrix is extremely serious and distributed in a nonuniform state across the composite.

In Figure 8, Figure 9 and Figure 10, the major failure modes of the specimens under transverse shear loading are kink band under the loading head, matrix cracking, debonding and delamination of the fiber–matrix interface, and fiber bundle fracture. The kink band is formed by the dislocation of the fiber and the degradation of the matrix under shear action, and the cracking of the fiber bundle is caused by bottom tension [38]. The tip of the crack is the stress concentration point, which promotes the development of the crack in the matrix. Once the crack reaches the fiber, the fiber is sheared at a certain angle, resulting in the interface debonding and delamination of the fiber and matrix or fiber bundle fracture [39]. Under transverse shear, the internal damage of a sample is largely caused by the damage to the fiber and yarn. This finding indicates that the axial yarn and the braiding fiber play a supporting role under transverse loading. The fracture morphology of the yarn on the shear fracture of the CaCb sample is neat. A crack develops along different planes—from top to bottom and from bottom to top. The crack is radial along the shear loading direction when the fiber line breaks, and yarn splitting is serious, with debonding and delamination occurring at the interface of the fiber matrix. Evidently, the axial and braided yarns in the samples are broken, indicating that crack development reaches the carbon fiber and resulting in the fracture of the carbon fiber bundle. The internal damage patterns of the KaCb sample after loading failure include axial yarn aramid and braided yarn carbon fiber fracture, matrix fracture, fiber bundle yield, and matrix debonding. The number of internal fiber fractures decreases, the debonding and delamination of the fiber–matrix interface increase, the transverse crack increases, and the material does not separate completely. In the crack development process, cracks are distributed along the axial yarn following a strip shape, and the bending deformation of the axial yarn causes cracks to develop laterally [40]. The difference between yarn stress and resin stress in the middle region of the shear band is considerable, which is the primary reason for interface cracking and crack growth [41]. The failure damages of the CaKb sample include fracture of the carbon fiber in the axial yarns, debonding and delamination of the aramid matrix interfaces in braided yarns, fracture of the resin, yield of the fiber bundle, and shedding of the matrix. Internal damage is concentrated near the shear zone of the material, and nearly no damage is found far from the loading zone. Cracks are distributed intermittently during their development process. The bending deformation of the braided aramid fiber causes the cracks to develop laterally, and the axial carbon fiber’s shear fracture is serious. Fracture causes crack growth along the fiber–matrix interface, reducing the capability of the material to redistribute load. An increase in fiber breaks leads to resin aging and ultimate failure [42]. In summary, the synergistic effect of the braided and axial yarns in the sample results in the better overall performance of the material. The bending deformation of the aramid fiber in the hybrid braided sample can effectively prevent the longitudinal development of the grain, cause cracks to distribute horizontally along the yarn path, and slow down stress localization in the loading area.

In Figure 11, Figure 12 and Figure 13, the major failure modes of the specimen under longitudinal shear loading are matrix cracking and fiber debonding, which are caused by the sliding friction between the fiber and the matrix [43]. The top surface of the longitudinal specimen is relatively smooth, but a few cracks are found between the fiber bundle and the surface resin. When the fiber–matrix interface can no longer bear the shear stress, the fiber will slip [44], resulting in fiber and matrix cracking. Matrix cracking is the initial failure mode of composite materials. A crack propagates longitudinally and extends to the bottom surface, destroying the bond between the fiber and the matrix, forming matrix fracture, and resulting in failure. The complete fracture of the CaCb specimen is divided into two parts. The internal failure is concentrated near the shear zone of the material, but nearly no damage is found far from the loading zone. The yarn with shear fracture is seriously broken, and a large part of the resin is shed. Evidently, the axial yarn in the sample slips toward both sides of the shear zone, the braided yarn breaks, and the resin cracks seriously. The damages of the KaCb specimen after longitudinal loading failure include resin cracking and fiber bundle yielding. The internal damage is concentrated in the area near the shear back of the material, the material is not completely separated, and no longitudinal cracks are found inside. After the failure of the CaKb sample under longitudinal loading, the resin is cracked seriously, and internal cracks are distributed along the braided yarns. The bending deformation of the braided yarn aramid in the sample is serious. When the specimen is loaded longitudinally, the axial yarn inside the specimen slides toward both sides, and the braided yarn and the matrix bear the load together. The fracture of the matrix is the primary cause of specimen failure. Given the good deformation capability and interface effect of aramid fiber, the hybrid braiding composite exhibits good shear resistance when the braiding yarn is aramid fiber.

## 4. Conclusions

The shear failure characterization of braided composites with pure carbon and carbon/aramid hybrid is investigated via short beam shear evaluation with different bearing load directions. The macro damage of the loaded samples is examined through optical microscopy and immersion ultrasound scanning. Meanwhile, the micro damage of the samples is investigated via µCT scanning. It has been found that the maximum load, shear strength, and energy absorption capacity of the specimens under transverse loading are higher than those under longitudinal loading because the axial yarn and braided fiber of the specimens under transverse loading share supporting roles. The shear toughness of hybrid braided composite has been improved to a certain degree compared with the pure carbon fiber composite under both transverse and longitudinal directions. The damage morphology also proves that the portions of fiber local damage and progressive damage have been raised due to the hybrid effect, thus extending the damage process. Besides, the different hybrid structure plays a varied role in controlling the shear toughness under transverse and longitudinal loadings. CaKb achieves the best shear toughness under transverse shear loading, but KaCb demonstrates better shear toughness than the other two. The aramid fiber produces a positive effect when acting as the axial yarn under transverse shear loading. The ductility of aramid fiber in the hybrid braided composite can effectively prevent the propagation of internal cracks under shear loading. The extensive interface influence of aramid fiber provides a synergistic effect on the composites, which is also the primary reason why the shear resistance of the composites is enhanced.

While carbon fiber provides the strength of the composites, the addition of aramid fiber at an appropriate position can further improve the toughness of the composites. Thus, the two fibers work together to improve the overall shear properties of hybrid composites. In order to reduce sudden failure risks and improve composite performance, hybrid braiding is an effective way to develop composite materials, and hybrid braided structure is promising in engineering applications.

## Figures and Tables

**Table 1:** Material parameters.

Materials	Type	Strength (MPa)	Modulus (GPa)	Density (g/cm^3^)	Elongation (%)
Carbon fiber	T300-3K	3530	230	1.76	1.5
Aramid fiber	Kevlar49	2923	95	1.44	2.8
Resin	TDE-86	80	3.5	1.13	4.0

**Table 2:** Composite specifications.

Type	Axial	Braid	Pick Length (mm)	Pick Width (mm)	Yarn Volume Content (%)
CaCb	T300-3K	T300-3K	25 ± 0.1	25 ± 0.1	T300-3K:53%
CaKb	T300-3K	Kevlar49	25 ± 0.1	25 ± 0.1	Kevlar49:11%, T300-3K:43%
KaCb	Kevlar49	T300-3K	25 ± 0.1	25 ± 0.1	Kevlar49:43%, T300-3K:11%

## References

[1] Gu Q., Quan Z., Yu J., Yan J., Sun B., Xu G. (2019). Structural modeling and mechanical characterizing of three-dimensional four-step braided composites: A review. Compos. Struct..

[2] Wu L., Zhang F., Sun B., Gu B. (2014). Finite element analyses on three-point low-cyclic bending fatigue of 3-d braided composite materials at microstructure level. Int. J. Mech. Sci..

[3] Luo H., Xiong G., Yang Z., Raman S.R., Li Q., Ma C., Li D., Wang Z., Wan Y. (2014). Preparation of three-dimensional braided carbon fiber-reinforced peek composites for potential load-bearing bone fixations. Part i. Mechanical properties and cytocompatibility. J. Mech. Behav. Biomed. Mater..

[4] Xiang D., Wang L., Tang Y., Harkin-Jones E., Zhao C., Wang P., Li Y. (2018). Damage self-sensing behavior of carbon nanofiller reinforced polymer composites with different conductive network structures. Polymer.

[5] Khosravani M.R., Weinberg K. (2017). Experimental investigations of the environmental effects on stability and integrity of composite sandwich t-joints. Mater. Und Werkst..

[6] Singh T.J., Samanta S. (2015). Characterization of kevlar fiber and its composites: A review. Mater. Today Proc..

[7] Wu L., Wang W., Jiang Q., Lin J.-H., Tang Y. (2020). Illustrating hybrid effect and damage evolution of carbon/aramid braided composite under low-velocity impact. Compos. Struct..

[8] Jawaid M., Abdul Khalil H.P.S., Abu Bakar A. (2011). Woven hybrid composites: Tensile and flexural properties of oil palm-woven jute fibres based epoxy composites. Mater. Sci. Eng. A.

[9] Swolfs Y., McMeeking R.M., Rajan V.P., Zok F.W., Verpoest I., Gorbatikh L. (2015). Global load-sharing model for unidirectional hybrid fibre-reinforced composites. J. Mech. Phys. Solids.

[10] Zheng Y., Sun Y., Li J., Limin L., Chen L., Liu J., Tian S. (2017). Tensile response of carbon-aramid hybrid 3d braided composites. Mater. Des..

[11] Wu L., Wang W., Jiang Q., Xiang C., Lou C.W. (2019). Mechanical characterization and impact damage assessment of hybrid three-dimensional five-directional composites. Polymers (Basel).

[12] Zhang P.-F., Zhou W., Yin H.-F., Shang Y.-J. (2019). Progressive damage analysis of three-dimensional braided composites under flexural load by micro-ct and acoustic emission. Compos. Struct..

[13] Zhang D., Waas A.M., Yen C.-F. (2015). Progressive damage and failure response of hybrid 3d textile composites subjected to flexural loading, part i: Experimental studies. Int. J. Solids Struct..

[14] Murugan R., Ramesh R., Padmanabhan K. (2014). Investigation on static and dynamic mechanical properties of epoxy based woven fabric glass/carbon hybrid composite laminates. Procedia Eng..

[15] Godani M., Gaiotti M., Rizzo C.M. (2014). Interlaminar shear strength of marine composite laminates: Tests and numerical simulations. Compos. Struct..

[16] Albahash Z.F., Ansari M.N.M. (2017). Investigation on energy absorption of natural and hybrid fiber under axial static crushing. Compos. Sci. Technol..

[17] He C., Ge J., Zhang B., Gao J., Zhong S., Liu W.K., Fang D. (2020). A hierarchical multiscale model for the elastic-plastic damage behavior of 3d braided composites at high temperature. Compos. Sci. Technol..

[18] Liu G., Zhang L., Guo L.C., Wang Q.M., Liao F. (2017). A modified v-notched beam test method for interlaminar shear behavior of 3d woven composites. Compos. Struct..

[19] Nayak R.K., Rathore D., Ray B.C., Routara B.C. (2017). Inter laminar shear strength (ilss) of nano al2o3 filled glass fiber reinforced polymer (gfrp) composite—A study on loading rate sensitivity. Mater. Today Proc..

[20] Xiao S., Wang P., Soulat D., Minet J., Zemni L., Gao H. (2018). Analysis of the in-plane shear behaviour of non-orthogonally textile reinforcements: Application to braided fabrics. Compos. Part B Eng..

[21] Ahmad M.A.A., Abdul Majid M.S., Ridzuan M.J.M., Mazlee M.N., Gibson A.G. (2018). Dynamic mechanical analysis and effects of moisture on mechanical properties of interwoven hemp/polyethylene terephthalate (pet) hybrid composites. Constr. Build. Mater..

[22] Gereke T., Cherif C. (2019). A review of numerical models for 3d woven composite reinforcements. Compos. Struct..

[23] Cui C., Dong J., Mao X. (2019). Effect of braiding angle on progressive failure and fracture mechanism of 3-d five-directional carbon/epoxy braided composites under impact compression. Compos. Struct..

[24] Ge J., He C., Liang J., Chen Y., Fang D. (2018). A coupled elastic-plastic damage model for the mechanical behavior of three-dimensional (3d) braided composites. Compos. Sci. Technol..

[25] Liu X., Zhang D., Sun J., Yu S., Dai Y., Zhang Z., Sun J., Li G., Qian K. (2020). Refine reconstruction and verification of meso-scale modeling of three-dimensional five-directional braided composites from x-ray computed tomography data. Compos. Struct..

[26] Zhou H., Li C., Zhang L., Crawford B., Milani A.S., Ko F.K. (2018). Micro-xct analysis of damage mechanisms in 3d circular braided composite tubes under transverse impact. Compos. Sci. Technol..

[27] Ya J., Liu Z., Wang Y. (2016). Micro-ct characterization on the meso-structure of three-dimensional full five-directional braided composite. Appl. Compos. Mater..

[28] Khosravani M.R. (2012). Composite materials manufacturing processes. Appl. Mech. Mater..

[29] Makeev A., He Y., Schreier H. (2013). Short-beam shear method for assessment of stress–strain curves for fibre-reinforced polymer matrix composite materials. Strain.

[30] Garcea S.C., Wang Y., Withers P.J. (2018). X-ray computed tomography of polymer composites. Compos. Sci. Technol..

[31] Meza L.R., Schormans J.M.J., Remmers J.J.C., Deshpande V.S. (2019). Shear response of 3d non-woven carbon fibre reinforced composites. J. Mech. Phys. Solids.

[32] Zhang D., Liu X., Gu Y., Sun M., Yu S., Zhang Y., Qian K. (2018). Effects of off-axis angle on shear progressive damage of 3d woven composites with x-ray micro-computed tomography. Compos. Part A Appl. Sci. Manuf..

[33] Tian Z., Yan Y., Li J., Hong Y., Guo F. (2018). Progressive damage and failure analysis of three-dimensional braided composites subjected to biaxial tension and compression. Compos. Struct..

[34] Lu Y. (2010). Non-Destructive Evaluation on Concrete Materials and Structures Using Cement-Based Piezoelectric Sensor. Ph.D Thesis.

[35] Segreto T., Bottillo A., Teti R. (2016). Advanced ultrasonic non-destructive evaluation for metrological analysis and quality assessment of impact damaged non-crimp fabric composites. Procedia CIRP.

[36] Aymerich F., Meili S. (2000). Ultrasonic evaluation of matrix damage in impacted composite laminates. Compos. Part B-Eng..

[37] Bull D.J., Helfen L., Sinclair I., Spearing S.M., Baumbach T. (2013). A comparison of multi-scale 3d x-ray tomographic inspection techniques for assessing carbon fibre composite impact damage. Compos. Sci. Technol..

[38] Dong C., Davies I.J. (2018). Effect of stacking sequence on the flexural properties of carbon and glass fibre-reinforced hybrid composites. Adv. Compos. Hybrid Mater..

[39] Wang R., Zhang L., Hu D., Liu X., Cho C., Li B. (2017). Progressive damage simulation in 3d four-directional braided composites considering the jamming-action-induced yarn deformation. Compos. Struct..

[40] Li Y., Sun B., Gu B. (2017). Impact shear damage characterizations of 3d braided composite with x-ray micro-computed tomography and numerical methodologies. Compos. Struct..

[41] Wan Y., Straumit I., Takahashi J., Lomov S.V. (2016). Micro-ct analysis of internal geometry of chopped carbon fiber tapes reinforced thermoplastics. Compos. Part A Appl. Sci. Manuf..

[42] Li X. (2012). Eddy Current Techniques for Non-Destructive Testing of Carbon Fibre Reinforced Plastic (CFRP).

[43] Huang N.C., Liu X.Y. (1994). Debonding and fiber pull-out in reinforced composites. Theor. Appl. Fract. Mech..

[44] Sebaey T.A., Catalanotti G., O’Dowd N.P. (2019). A microscale integrated approach to measure and model fibre misalignment in fibre-reinforced composites. Compos. Sci. Technol..

